# scVAR: integrating genomics and transcriptomics from single-cell RNA-seq —insights from leukemia case studies

**DOI:** 10.3389/fgene.2025.1604484

**Published:** 2026-01-05

**Authors:** Ludovica Celli, Samuele Manessi, Matteo Barcella, Ivan Merelli

**Affiliations:** 1 Institute of Biomedical Technologies, National Research Council (ITB-CNR), Segrate, Italy; 2 Bioinformatics Core, San Raffaele Telethon Institute for Gene Therapy, IRCCS San Raffaele Scientific Institute, Milan, Italy

**Keywords:** genetic heterogeneity, leukemia, multi-omics integration, single-cell RNA sequencing, variational autoencoder

## Abstract

The advent of high-throughput technologies has accelerated biomedical research by facilitating the investigation of biological complexity at unprecedented resolution. Single-cell RNA sequencing (scRNA-seq) has transformed our ability to deconstruct cellular heterogeneity in complex diseases. Acute myeloid leukemia (AML) and acute lymphoblastic leukemia (ALL), for example, are characterized by extensive genetic and phenotypic heterogeneity, making diagnosis and therapy challenging. Although genetic variation is conventionally studied via DNA-based methods, the transcriptome can also be a source of genomic information. Here, we present scVAR, a computational framework that employs variational autoencoders to learn and integrate genetic variation directly from scRNA-seq data. scVAR implements a paired encoder–decoder architecture with a cross-attention–based fusion layer that combines transcriptomic and variant-derived information into a unified latent representation, enhancing the detection of subtle cellular differences under noisy and sparse conditions. We demonstrate its application to leukemia case studies, where scVAR reveals cell identities that are not discernible when transcriptomic or genomic data are analyzed separately. In the datasets analyzed in this study, scVAR identifies approximately 20%–30% more subpopulations than transcriptomic analysis alone, highlighting the benefit of integrating variant information even when coverage is limited. As expected for 3′ scRNA-seq, variant detection is restricted to captured regions, but scVAR maximizes the information available within these constraints. Overall, scVAR bridges the gap between transcriptomics and genomics, providing a broadly applicable platform for the integrative characterization of cell states and disease processes.

## 1 Introduction

Over the past two decades, the rapid development of high-throughput technologies has revolutionized our ability to explore biological complexity. Omics disciplines such as genomics and transcriptomics have driven the identification of key molecular pathways and mechanisms underlying various diseases, which in turn has supported the development of novel therapeutic strategies (Zhang et al., 2011; Fan et al., 2020). Among these advancements, single-cell RNA sequencing (scRNA-seq) (Chen et al., 2019) has transformed our understanding of cellular heterogeneity, particularly in complex diseases such as hematological malignancies (Navin et al., 2010; Miles et al., 2020).

Leukemia, for instance, is a group of highly heterogeneous blood cancers, and exemplifies the complexity that scRNA-seq can help dissect (Liu et al., 2024). Acute myeloid leukemia (AML) and acute lymphoblastic leukemia (ALL) are the two most aggressive subtypes, originating in the bone marrow. AML is characterized by the production of more than 20% of immature cells (blasts) that give rise to the myeloid lineage of the blood cells. This abnormal production of myeloblasts often depends on gain or loss of chromosomes, chromosomal rearrangements, and translocations, such as t (15; 17) (Menghrajani et al., 2020; De-Morgan et al., 2021). ALL, on the other hand, is characterized by the same proportion of immature lymphoid cells that normally differentiate into B- or T-cell lymphocytes. Similarly, in ALL, abnormal chromosome numbers or translocations can lead to the production of lymphoblasts, with the most common mutations including t (12; 21) and t (9; 22) (Tran and Hunger, 2022).

Thus, despite differences in terms of affected cells, mutations and markers, leukemias are characterized by substantial genetic and phenotypic diversity. This heterogeneity poses significant challenges for diagnosis, prognosis, and therapeutic intervention. Such complexity arises from a multitude of biological factors, with the presence of clones bearing distinct genetic profiles being one of the most significant drivers (Schwede et al., 2024). While genetic variation is typically studied using DNA-based technologies, such as Whole Exome Sequencing (WES) and Whole Genome Sequencing (WGS), the transcriptome also contains valuable genomic information (Rabbani et al., 2014). Tools developed for bulk RNA-seq have shown that genetic variants can be extrapolated from transcriptomic data, and this concept extends to scRNA-seq, enabling variant inference at single-cell resolution (Harmanci et al., 2022; Shafighi et al., 2021; Vu et al., 2019; Tickle et al., 2019).

The fact that both genetic and transcriptomic informations can be assessed in a single assay can strongly limitate confounding factors. Such integration is critical for enhancing the accuracy of identifying genotype–phenotype correlations in diseases like leukemia and for uncovering the interactions between genomic and transcriptomic layers that drive cellular behavior (Tordini et al., 2016).

Integrating genomic and transcriptomic signals from single-cell data remains computationally challenging, and several strategies have been proposed to tackle multi-omic integration. Common approaches include Principal Component Analysis (PCA) concatenation, which merges reduced features from each modality; Weighted Nearest Neighbors (WNN), which integrates cell–cell similarity graphs using modality-specific weights; and Multi-Omics Factor Analysis (MOFA/MOFA+), which learns shared and modality-specific latent factors (Hao et al., 2021; Argelaguet et al., 2018; Argelaguet et al., 2020). These frameworks have proved effective for paired multi-omic datasets, but they generally require balanced modalities or explicitly measured multi-layer profiles. In contrast, scRNA-seq–derived variant information is sparse, unevenly distributed, and highly dependent on gene expression, making direct application of these methods suboptimal. This motivates the development of an approach specifically designed to integrate transcriptomic and variant-derived signals obtained from scRNA-seq alone.

To address these challenges, we developed scVAR, a computational tool designed to infer and integrate genetic information directly from scRNA-seq data. By leveraging advanced neural network architectures such as variational autoencoders, scVAR simultaneously analyzes genetic and transcriptomic heterogeneity, offering a comprehensive view of cellular diversity. Its design specifically addresses the sparse coverage inherent to scRNA-seq protocols, enabling the detection of meaningful variants and providing a robust vertical integration (Argelaguet et al., 2021) of variant- and expression-derived representations.

In this study, we apply scVAR to both AML and B-ALL single-cell RNA-seq datasets produced with the 10X Genomics technology (3′ and 5′ kit, respectively).

- a B-cell acute lymphoblastic leukemia (B-ALL) dataset (n = 3 diseases)
- and an acute myeloid leukemia (AML) dataset (n = 2 diseases)

This technology reduces the number of detectetable single-nucleotide variants (SNVs) because it primarily captures Untranslated Regions (UTRs), thus representing a limitation in the depth of genomic characterization. Nevertheless, genetic variants at UTRs may modify regulatory elements, which can in turn result in transcriptional modulation (Steri et al., 2018). Moreover, the scope of this work is to understand how the genomic and transcriptomic layers interact to shape the integrated space, rather than to identify pathogenetic variants. Through this application, we illustrate how scVAR helps in the identification of cellular subpopulations whose unique characteristics may arise only in the integrated modalities, thus uncovering hidden biological insights.

The results presented herein highlight the potential of scVAR to bridge the gap between genomics and transcriptomics in scRNA-seq. By integrating these layers of information, scVAR enhances our understanding of leukemia biology and establishes a broadly applicable framework for dissecting complexity in other diseases. This paper describes the underlying methodology of scVAR, its implementation, and its application to AML (Naldini et al., 2023) and B-ALL (Caserta et al., 2023) scRNA-seq datasets, and provides guidance for data interpretation.

## 2 Materials and methods

**FIGURE 1:**
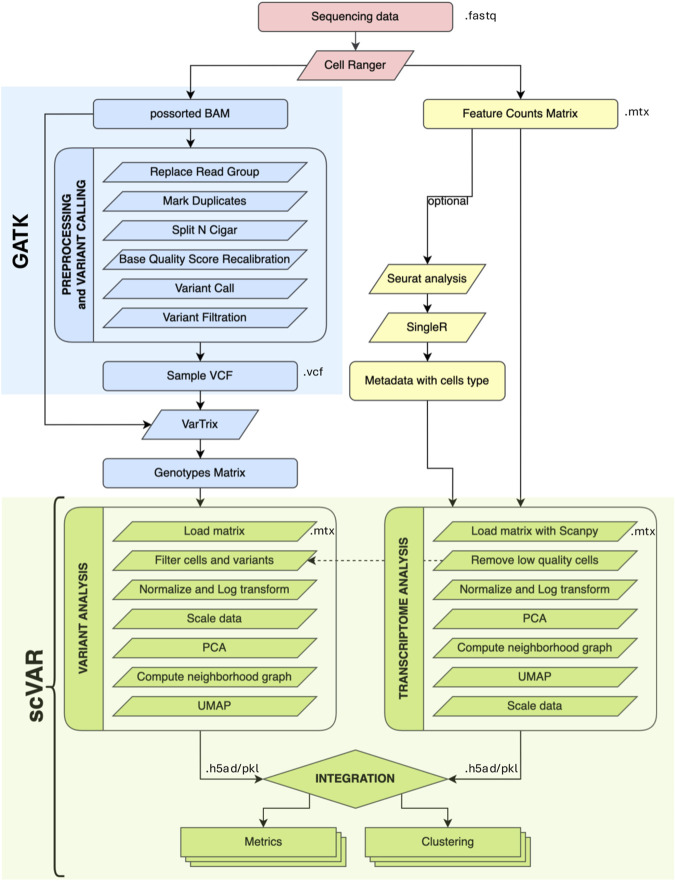
Overview of the scVAR pipeline. Raw sequencing data is processed using the Cell Ranger pipeline, followed by preprocessing of the alignment file to obtain high-quality reads for variant calling. The VarTrix tool is then used to create a matrix describing the genotype of each notable locus in each cell. scVAR performs both variant and transcriptomic analyses by integrating this matrix with the feature count matrix from Cell Ranger. Finally, the results are combined to generate a new dimensional reduction that captures both transcriptomic and genomic data. Additionally, scVAR can perform clustering and calculating various metrics for each of the provided representations. Color code: pink indicates steps performed through Cell Ranger; blue indicates steps performed using GATK while green is for the analyses performed through scVAR. Input and output file formats are indicated in the figure.

This section describes the complete scVAR analysis workflow, including preprocessing, transcriptomic- and variant-level analyses, and the final integration of the two modalities (Figure 1). The scVAR workflow is organized into four main stages. It begins with the preprocessing of raw sequencing data through the Cell Ranger pipeline (v.7.1.2, 10x Genomics), which performs alignments and generates gene count matrices. Next, the workflow includes single-cell transcriptome analysis for quality control, normalization, and dimensionality reduction, followed by single-cell variant analysis to infer genomic variations directly from scRNA-seq reads. Finally, scVAR performs the integration of the two omic layers, combining transcriptomic and genomic information within a unified latent representation (Figure 1).

### 2.1 Preprocessing: raw data quality check, alignment and count matrix generation

Both AML (3′ kit) and B-ALL (5′ kit) scRNA-seq datasets were obtained using the Chromium 10x platform and sequenced on the Illumina NextSeq or NovaSeq platform. The Cell Ranger *count* pipeline was applied to perform reads alignment to the human reference genome (GRCh38) using STAR (2.7.2a) and to generate BAM files (possorted BAMs) and gene expression matrices. Quality control metrics, including sequencing saturation and fraction of reads mapped to exons, were computed as part of the pipeline and reported in the Cell Ranger web summary. The resulting sparse matrices were then used for downstream analyses.

### 2.2 Genomic-level analysis

The BAM files obtained from the Cell Ranger pipeline were used to perform the variant calling and to generate a matrix describing the variants per cell, to obtain a genetic-based representation.

#### 2.2.1 Variant calling from scRNA-seq reads

A new variant-calling pipeline was implemented following the GATK Best Practices workflow for RNA-seq short variant discovery (Van der Auwera et al., 2013). According to GATK guidelines, all reads were assigned to a uniform Read Group (RG) using Picard’s *AddOrReplaceReadGroups*. Duplicate reads were then marked using MarkDuplicates, although this step is generally redundant for 10x Genomics possorted BAM files because PCR duplicates are already identifiable through UMIs.

To ensure correct alignment across splice junctions, reads were processed with GATK’s *SplitNCigarReads*, which splits reads at intron–exon junctions and adjusts CIGAR strings accordingly. High-confidence variant calling was further supported by Base Quality Score Recalibration (BQSR), performed using GATK v4.2.0.0. In particular, *BaseRecalibrator* was used to model systematic errors in base quality scores based on multiple covariates (including read group, reported quality, machine cycle, and nucleotide context) and to generate a recalibration table. This table was then applied with *ApplyBQSR* to update base qualities prior to variant calling.

Variant calling was performed using GATK *HaplotypeCaller*, which was chosen for its improved accuracy in difficult-to-call genomic regions and superior performance in detecting insertions and deletions (indels) compared to position-based callers such as *UnifiedGenotyper* (Poplin et al., 2018; Quinones-Valdez et al., 2022) and it properly handles spliced alignments. The minimum phred-scaled confidence threshold for variant calling was set to 20, as suggested by the documentation and a Variant Call Format (VCF) file was obtained for each dataset. Low-quality variants were removed using a hard-filtering approach with GATK *VariantFiltration*. Variants were filtered using a window size of 35 bases and a cluster threshold of 3, which means that any region containing three or more variants within a window of 35 nucleotides was flagged for removal. Additional filters were applied to remove variants with a Fisher Strand (FS) score greater than 30, QualByDepth (QD) lower than 2, and Depth (DP) lower than 100. To refine genotype-level filtering, we excluded genotypes with DP lower than 30 and Genotype Quality (GQ) lower than 50. All other parameters were kept at their default values. After this filtering step, bcftools view was used to retain only variants labeled as PASS (Danecek et al., 2021).

To infer genetic information at the single-cell level, we used VarTrix (v1.1.22, 10x Genomics), which assigns variants to individual barcodes by evaluating reads already aligned in the Cell Ranger BAM at the variant loci defined in our filtered VCF files. The tool relies on the existing read alignment and associated CIGAR/MD tags, without performing any additional realignment. We ran VarTrix in consensus mode, enabling UMI-aware processing and restricting the analysis to primary alignments. A padding of 200 bases was applied to extend the genomic window around each locus, ensuring that all reads overlapping the variant site were considered. VarTrix reports, for each variant–barcode pair, the number of reads supporting the reference and alternative alleles, and was configured to call genotypes only when a locus was covered by at least three reads in a given cell (default behaviour). The resulting variant-by-barcode matrix encodes per-cell genotypes as 0 (insufficient coverage), 1 (reference homozygous), 2 (heterozygous) or 3 (alternative homozygous), and was subsequently imported into scVAR for downstream analyses.

#### 2.2.2 scVAR–genomic analysis module

scVAR automatically imports and processes the variant matrix generated by VarTrix in a manner similar to the transcriptomic data. Using the AnnData object from the transcriptome analysis and the variant matrix file, scVAR loads the data and matches barcodes between the two omic layers. Any cell present in the variant matrix but not in the transcriptomic AnnData object is removed to ensure consistency, as transcriptomic quality control may have excluded some barcodes. A minimum-cell-fraction threshold can be applied to remove variant loci detected in too few cells, after which the matrix is standardized by centering to zero mean and scaling to unit variance. Principal components (PCs) and nearest neighbors (NNs) are computed to obtain a Uniform Manifold Approximation and Projection (UMAP) representation that captures relationships based solely on genomic variation. The processed data and derived embeddings are stored in the AnnData object for downstream analyses.

### 2.3 Transcriptomic-level analysis

The standard analysis workflow using Cell Ranger and Scanpy (Wolf et al., 2018) was applied for transcriptomic processing. Further details are provided in the sections below.

#### 2.3.1 scVAR–transcriptomic analysis module

The counts matrices were used as input to analyze the transcriptome at single-cell resolution, in a unique function. Cells were filtered out if expressing fewer than 200 genes or if the percentage of mitochondrial genes was greater than 12%. Additionally, genes expressed in fewer than 3 cells were not included in the downstream analyses. Counts per cell were normalized and a natural log transformation of the data matrix was performed. Genes having mean expression between 0.015 and 3 and dispersion over 0.75 were considered highly variable.

Next, PCA was performed for defining the top PCs to use for the subsequent neighborhood graph computation. Lastly, UMAP was run on the neighborhood graph to generate a low-dimensional embedding that preserves local transcriptomic relationships. Finally, features were standardized by centering to zero mean and scaling to unit variance, storing this result for the following integration. In all the datasets presented in this manuscript the top 30 PCs were used.

#### 2.3.2 Metadata integration

The datasets were further enriched by integrating cell type annotations obtained through previous transcriptomic analyses. Specifically, Seurat v4.0.3 was used to perform clustering on the transcriptomic data, while SingleR was applied to assign cell types based on a reference database (Blueprint/ENCODE). The resulting metadata was incorporated into the scVAR software for visualization and analysis. This part of the transcriptome analysis is optional, as it is performed outside the scVAR modules, and can be used as input for the transcriptome module to enrich the information.

### 2.4 Integrated-level analysis (Genomic + transcriptomic)

scVAR integrates transcriptomic and genomic single-cell data through a paired autoencoder architecture designed to learn a shared latent representation of both modalities in an unsupervised manner. Rather than concatenating principal components (PCA concatenation), scVAR employs two modality-specific encoders and decoders that process transcriptomic and variant-derived features separately while enforcing a common low-dimensional manifold that captures their coordinated structure. Each encoder first projects its respective omic layer into a latent space through a series of nonlinear transformations with batch normalization and dropout regularization. The resulting embeddings are then combined through a fusion layer using a local cross-attention mechanism: for each cell, the transcriptomic latent vector interacts with the k most similar genomic vectors (top-k attention), weighted by their cosine similarity. A sigmoid gating function adaptively balances contributions from the two omic views, yielding a fused latent embedding that integrates gene expression and variant information in a context-aware manner.

Optionally, a multi-head self-attention block acts on the fused embedding to refine global structure and improve representation consistency across cellular states. The model is trained end-to-end by minimizing a composite loss function that combines (i) omic-specific reconstruction errors, (ii) cross-reconstruction between the two domains, (iii) alignment loss combining mean-squared and contrastive terms (SimCLR-style) (Chen et al., 2020), and (iv) mild regularization on latent variance and cosine similarity. This multi-term objective enforces both the preservation of modality-specific information and the convergence of the two latent manifolds.

After training, the encoders generate embeddings for each omic layer (zA for transcriptomics, zB for genomic variants), which are averaged into a final integrated latent space (zAE). This shared representation is then used for downstream analyses, including neighborhood graph construction and Leiden clustering, providing a coherent view of cellular heterogeneity across genomic and transcriptomic layers. Through this architecture, scVAR achieves a biologically grounded and noise-robust integration of single-cell multi-omics data, enabling the discovery of relationships between genetic variation and transcriptional state at single-cell resolution.

### 2.5 Clustering analysis

Clustering was performed using Scanpy, both on scRNAseq dataset alone and leveraging the precomputed integrated latent representation. The user selects an omics dataset, and scVAR constructs a neighborhood graph based on a specified number of PCs and neighbors. Clusters are then identified using the Leiden algorithm (Traag et al., 2019), with an adjustable resolution parameter. Higher resolution values yield more clusters, typically ranging between 0 and 2, depending on the dataset. Cluster assignments are stored as metadata and can be visualized across multiple UMAP representations.

### 2.6 Variant heatmap visualization

To visualize the distribution of genetic variants across cells, a heatmap can be generated using the ComplexHeatmap package in R. This heatmap displayed the variant data in a matrix format, with rows representing variant loci and columns representing cells. Cells were clustered based on their variant profiles, and the heatmap displays reference/alternative genotypes as defined by VarTrix.

### 2.7 Synthetic dataset generation and benchmarking

To evaluate the robustness and accuracy of scVAR under controlled and reproducible conditions, we developed a custom *in silico* generator capable of producing paired single-cell datasets that include both transcriptomic and genomic information for each simulated cell.

The generator was designed to mimic the statistical properties and noise patterns typical of real scRNA-seq data, while maintaining full knowledge of the underlying ground truth. Each synthetic dataset is composed of two coupled matrices, representing gene expression and variant information, generated according to predefined cell types and genotypes. For each cell type, a characteristic gene expression program is sampled and then perturbed by random deviations to reproduce realistic biological variability. Similarly, variant profiles are created for each genotype and subject to stochastic fluctuations in the number and distribution of alleles, emulating the limited and uneven coverage that characterizes single-cell sequencing data.

To further challenge the integration methods, the simulation can introduce mismatches between modalities, such as random label swaps between transcriptomic and genomic layers, as well as variable degrees of sparsity and dropout. The overall noise level can be tuned by adjusting the amplitude of these perturbations. Using this framework, we generated a panel of six datasets ranging from 5,000 to 50000 simulated cells, covering different sizes and noise conditions. These data were then used to compare the integration performance of scVAR with three commonly adopted multi-omic methods implemented in MUON (Bredikhin et al., 2022): PCA concatenation, which merges the principal components of each modality; WNN, which integrates cell–cell similarity graphs based on modality-specific weights; and MOFA, a latent factor model that jointly captures shared and specific sources of variability (Hao et al., 2021; Argelaguet et al., 2018; Argelaguet et al., 2020).

All methods were trained on identical input matrices using the same normalization, dimensionality, and clustering parameters to ensure a fair comparison. To quantify performance, the embeddings produced by each integration approach were clustered with the Leiden algorithm, and the resulting cluster assignments were compared with the known true labels defined during data simulation.

The degree of correspondence was measured using the Adjusted Rand Index (ARI), a standard metric of clustering agreement against the ground-truth labels. Execution time and model convergence were also recorded to evaluate computational efficiency. This benchmarking strategy was not intended to provide an exhaustive ranking of existing tools, but rather to test whether the variational autoencoder architecture adopted in scVAR could maintain accuracy and stability across heterogeneous datasets while scaling efficiently to larger data volumes.

### 2.8 Computational performance

To provide practical guidance for users and address reproducibility, we evaluated the computational performance of scVAR across the same synthetic datasets used in the benchmarking analysis. All runs were performed on a server equipped with 2× Intel Xeon Gold 6,252 CPUs (2.10 GHz), 1.5 TB RAM and one NVIDIA A100 40 GB GPU. Runtime scaled approximately linearly with dataset size, ranging from ∼30 s for 5,000 cells to ∼6–8 min for 50000 cells, including data loading, model training, and latent space generation. GPU acceleration reduced training time by ∼40–50% compared with CPU-only execution. Memory usage remained modest, never exceeding 10 GB for the largest dataset. Although these values depend on hardware and specific preprocessing steps, they provide an indicative overview of the typical computational footprint of scVAR and confirm that the method is compatible with standard GPU-enabled servers commonly used for single-cell analysis.

## 3 Results

Before analysing patient samples, we first evaluated scVAR on synthetic datasets that mimic the sparsity, dropout and cross-modal discordance typical of single-cell data. These tests confirmed that the model can integrate transcriptomic and genomic information in a stable way across different noise and complexity levels. We then applied scVAR to scRNA-seq datasets from AML and B-cell acute B-ALL to assess whether the joint representation of gene expression and genetic variation provides additional biological resolution. Across both diseases, scVAR increased the number of identifiable subpopulations by roughly 20–30 percent compared with transcriptomic analysis alone, showing that integration can reveal data structures that would otherwise remain hidden.

### 3.1 Validation on synthetic data

To evaluate scVAR under controlled conditions, we generated a panel of *in silico* single-cell datasets designed to capture the main technical challenges of variant detection from scRNA-seq: sparsity, uneven coverage, dropout and cross-modal discordance. Each dataset included paired transcriptomic and genomic features with known ground-truth labels, allowing a direct assessment of clustering performance. We produced six datasets ranging from 5,000 to 50,000 cells under two noise regimes, corresponding to theoretical ARI ceilings of about 0.9 (low noise) and 0.7 (high noise).

We benchmarked scVAR against three representative integration methods implemented in MUON: PCA concatenation, WNN and MOFA (Hao et al., 2021; Argelaguet et al., 2018; Argelaguet et al., 2020). All methods were evaluated under identical preprocessing, dimensionality reduction and clustering settings to ensure comparability. The results revealed three consistent and biologically meaningful trends.

First, under low-noise conditions, MOFA achieved the highest ARIs, typically approaching the theoretical performance ceiling (≈0.88–0.90). In this regime, PCA concatenation and scVAR performed similarly, ranging between 0.83 and 0.87, indicating that when cross-modal correspondence is strong and the structure of the data is relatively linear, both methods successfully capture the underlying cellular organization. WNN systematically produced lower accuracy, reflecting its known sensitivity to modality-specific sparsity.

Second, as noise increased to ∼30%, the relative performance patterns shifted markedly. In this more challenging setting, scVAR demonstrated improved robustness: its ARI values typically ranged between 0.66 and 0.71, matching or slightly exceeding MOFA (≈0.64–0.67) across dataset sizes, while PCA concatenation and WNN degraded more substantially. This behaviour is consistent with the modelling principles underlying scVAR: the variational autoencoder architecture can smooth inconsistencies between modalities and leverage shared structure, making it well suited for scenarios in which variant calls are noisy or partially missing.

Third, scVAR displayed a broader performance range across simulations than classical approaches. While some configurations performed more modestly, well-tuned models achieved ARIs close to the upper bound allowed by the simulated noise. Rather than being a limitation, this variability reflects the flexibility of neural architectures: users familiar with tuning latent dimensionality, regularization strength or modality-weighting parameters can often extract substantially improved representations, particularly in high-complexity settings.

Overall, these simulations show that scVAR performs on par with standard integration strategies in clean, low-noise scenarios and becomes competitive—or advantageous—as sparsity, noise and dataset size increase. These conditions closely reflect real-world scRNA-seq variant datasets, where the genomic modality is often the noisiest component. This behaviour supports the use of scVAR for the analysis of AML and B-ALL samples, where the transcriptomic–genomic correspondence is inherently weak, and the data is highly sparse.

### 3.2 scVAR provides new insights into cell variability in AML

We first applied scVAR to scRNA-seq samples derived from AML patients PT07 and PT08 (GSE185993; Naldini et al., 2023). In particular, day 14 post chemotherapy sample from PT07 (PT07 - D14), day 30 post chemotherapy sample from PT08 (PT08 – D30) and two samples from PT08 relapses, the first with temporary response (PT08 – REL), whereas the latter without response (PT08 – RELNR).

**FIGURE 2:**
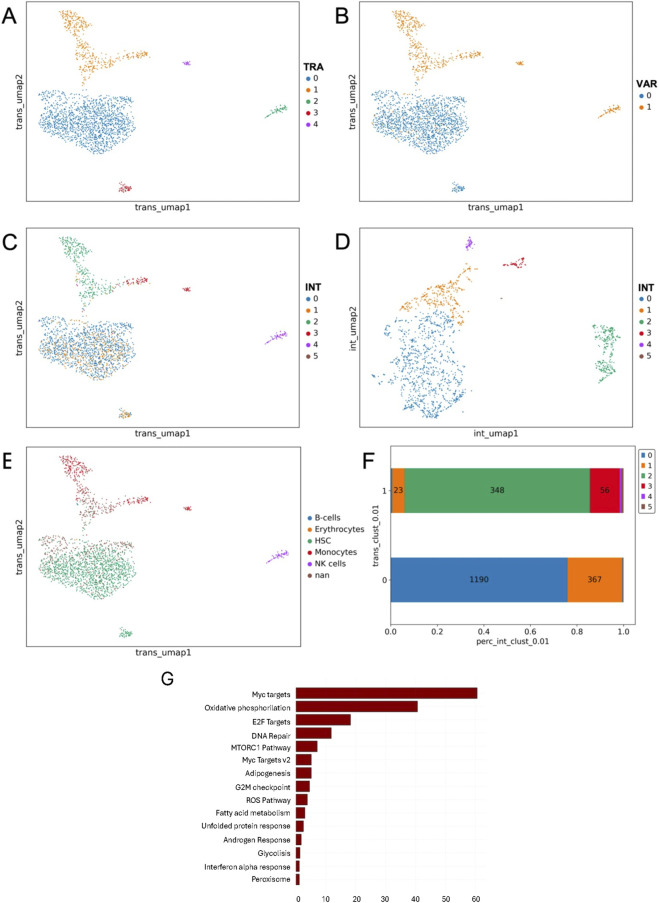
Sample from PT08 at day 30 post-chemo. **(A)** UMAP representation of transcriptomics clusters. **(B)** Transcriptomic UMAP representation of variant clusters over. **(C)** Transcriptomic UMAP representation of integrated clusters. All clustering methods **(A–C)** were performed with the same resolution. **(D)** UMAP of the integrated clusters, highlighting how the integration method resolves differences between clusters 0 and 1. **(E)** UMAP of transcriptomics clusters annotated using SingleR, with cells colored according to their predicted cell types. **(F)** Distribution of integrated clusters across the two main transcriptomics clusters. The y-axis represents transcriptomics clusters, while the x-axis shows the percentage of cells within each transcriptomics cluster, broken down by integrated clusters (indicated by different colors). The numbers above each bar represent the number of cells in each integrated cluster. **(G)** Barplot of significant hallmarks from enrichment analysis. Results are sorted by *−log10(p-value).*

In PT08-D30 sample, transcriptomic analysis identified five clusters (Figure 2A), while variant-based clustering spotted only two (Figure 2B), suggesting a higher heterogeneity driven by gene expression, rather than genetic variability. Indeed, at the same resolution, variant cluster 0 corresponds to transcriptomics cluster 0 and 3, while variant cluster 1 correspond to transcriptomic clusters 1,2 and 4 (Figures 2A,B). Integrating the two modalities with scVAR resulted in six clusters (Figure 2D), revealing additional subpopulations that were not detectable when projecting the integrated clusters on transcriptomic-driven embedding (Figure 2C). To evaluate the impact of each omic to the definition of integrated clusters, we analyzed their distributions across transcriptomics clusters (Figure 2F). For instance, transcriptomics cluster 0 is split in integrated cluster 0 (1,190 cells) and cluster 1 (367 cells). Additionally, integrated cluster 2 and 3 correspond to transcriptomic cluster 1 (Figure 2C), indicating that integration refines the resolution of transcriptomic variability. To further investigate the biological significance of the integrated clusters, we applied SingleR for cell type annotation. Interestingly, the hematopoietic stem cells (HSC) annotation corresponds to integrated clusters 0 and 1 (Figure 2E), highlighting that integration enhances functional interpretation. Differential gene expression analysis was run to elucidate the biological differences between the integrated clusters 0 and 1 within the transcriptomic cluster 0, annotated as HSC cells by SingleR. This analysis revealed 3,828 significant differentially expressed genes (DEGs), which were further examined through functional enrichment analysis spotting hallmarks such as Myc targets that can be associated with increased cell proliferation and tumor progression (Figure 2G).

For D14 sample from PT07, transcriptomics analysis defined three clusters (Supplementary Figure S1A), while variant data failed to provide meaningful subgroups (Supplementary Figure S1B). However, after performing scVAR integration, six distinct clusters were identified (Supplementary Figure S1D). The integrated clusters did not overlap significantly with the transcriptomics clusters (Supplementary Figure S1C), indicating that the integration revealed additional heterogeneity within the sample that was not apparent in the transcriptomics alone. Furthermore, increasing the resolution of the transcriptomics clustering did not reproduce the same clusters as those found in the integrated data. In the heatmap of the variants (Supplementary Figure S1E), we observe that the variants and cells are clustered together, showing similarities in their genotypes, and further emphasizing the importance of the integrated data in uncovering the underlying biological variability. This finding is quite in line to the biology of this sample that was basically free from leukemic blasts (Naldini et al., 2023). The genomic background is indeed similar to all the cells as expected from a healthy specimen.

**FIGURE 3:**
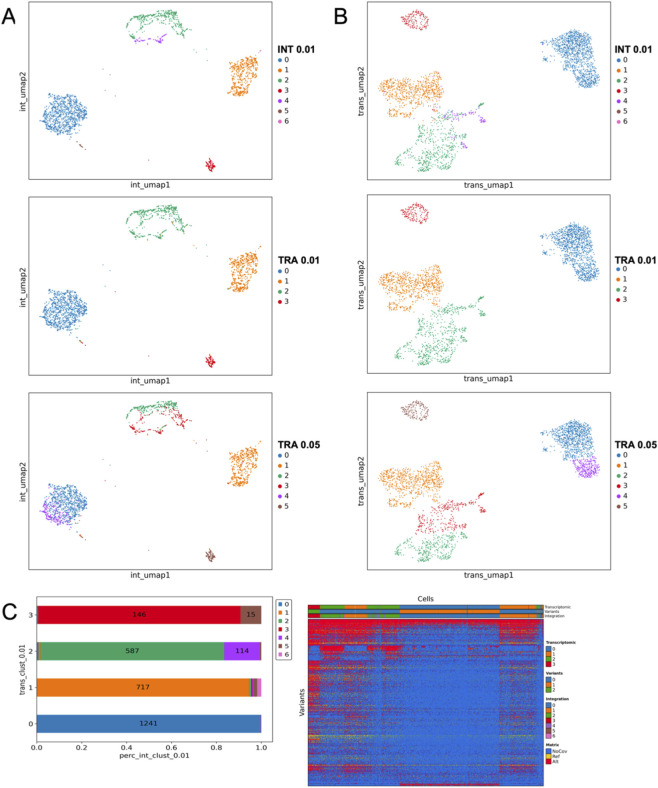
Sample PT08 at first relapse. **(A)** Integrated representation with cells colored by integrated clusters, showing transcriptomics clusters at resolutions 0.01 and 0.05. **(B)** Transcriptomics representation with the same clusters as in panel A, displaying both integrated and transcriptomics clusters at different resolutions. **(C)** The stack plot visualizes the distribution of integrated clusters across transcriptomics clusters, while the heatmap illustrates the distribution of clusters based on variant coverage (NoCov, no coverage variants; Ref, reference allele; Alt, Alternative allele).

Differently from previously described cases, in the following two case studies (PT08 – REL; PT08 REL NR) scVAR added little information. In the first relapse (Figure 3), transcriptomic and integrated clusters largely overlapped, except for integrated clusters 2 and 4, which subdivided transcriptomic cluster 2 (Figure 3A). Differently from PT08 -D30 and PT07- D14 samples, the integrated clusters are not well-mixed or overlapping, suggesting that the integration does not add new information. Increasing the transcriptomic resolution recapitulated nearly all integrated clusters, indicating that integration did not reveal new structure (Figure 3A). For each transcriptomic cluster, we observed an almost one-to-one correspondence with an integrated cluster, with only minor refinements introduced by variant information. The genotype heatmap showed distinct red–blue patterns driven mainly by coverage (blue): since scRNA-seq captures only expressed and sequenced variants, the detectable variant set shifts with gene expression (Figure 3C, right). As a result, variant-based clustering closely mirrors transcriptomic structure, limiting the additional contribution of integration in this sample (Figure 3C, left). A similar pattern is observed in PT08 – REL NR where both integrated and transcriptomic clustering reveal three main regions. In contrast, variant-based clustering produced only two groups driven by coverage. Integrated clusters overlapped almost perfectly with transcriptomic ones, showing that gene expression alone explained the structure of the sample (Supplementary Figure S2). In AML, relapsed disease typically exhibits a reduction in intratumoral genetic heterogeneity compared to diagnosis, characterized by the expansion of one or few dominant clones harboring stable driver mutations, although subclonal diversification may still occur in some cases reflecting ongoing clonal evolution under therapeutic pressure (Rapaport et al., 2021).

Together, these results indicate that scVAR increases resolution when transcriptomic and genetic layers provide complementary information, as seen at diagnosis and early post-treatment. At relapse, however, reduced clonal diversity and coverage-dependent variant detectability limited the added value of integration.

### 3.3 scVAR can unravel hidden variability in B-ALL samples at diagnosis

We next applied scVAR, following the same approach used above, to B-ALL samples. The full dataset includes samples from five patients (GSE236142; Caserta et al., 2023). In this work we applied the scVAR pipeline to three disease in which both diagnosis and relapse samples were available (L2, L5, L7) and their corresponding relapse samples (L4, L6, L8). As supporting metadata to interpret the results in B-ALL disease we have mainly B-cells, hence we could not include the annotation variable as in AML diseases. Conversely we exploited the miRNA-126 signature, whose expression is associated to more aggressive and chemo-resistant leukemic blasts.

**FIGURE 4:**
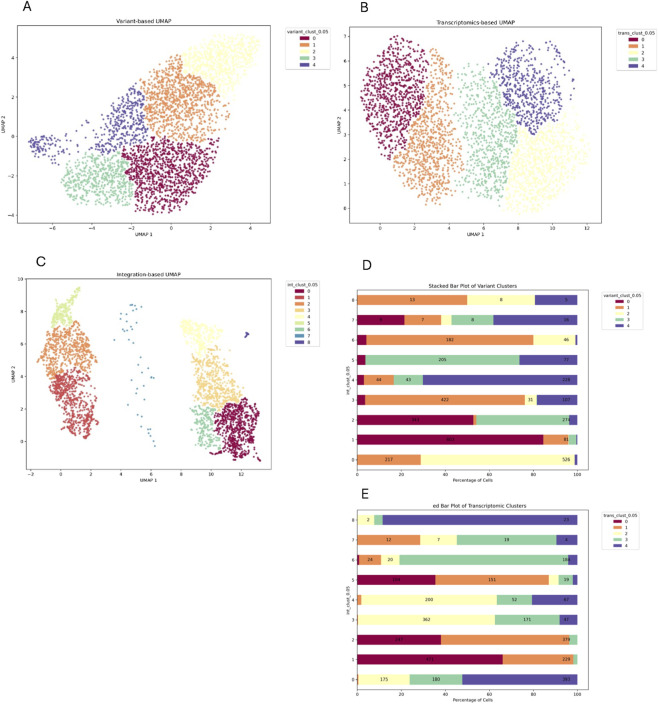
Sample L2. **(A)** Variant UMAP with cells colored by variant clusters. **(B)** Transcriptomic UMAP with cells colored by transcriptomic clusters. **(C)** Integrated UMAP with integrated clusters. The stacked barplot shows the integrated clusters distribution across variant **(D)** and transcriptomics clusters **(E)**. The y-axis represents genomics transcriptomics clusters respectively, while the x-axis shows the percentage of cells within each transcriptomics cluster, broken down by integrated clusters (indicated by different colors).

In sample L2, the variant- and transcriptomic-based UMAP defined both five clusters, suggesting an overall more balanced contribution to the heterogeneity of the samples (Figures 4A,B). scVAR integration not only defined nine clusters but clearly divided the UMAP into two spatially distant groups: (Figure 4C). With the exception of integrated cluster 8, which largely corresponded to transcriptomic cluster 0, transcriptomic- and variant-based clusters did not overlap with integrated ones, indicating that scVAR captured latent structure not detectable from individual modalities. Additionally, since the dataset is composed by miR-126-high and -low cells we tried to check if the integration follows signature expression, but unfortunately the overall expression was very low in this sample.

**FIGURE 5:**
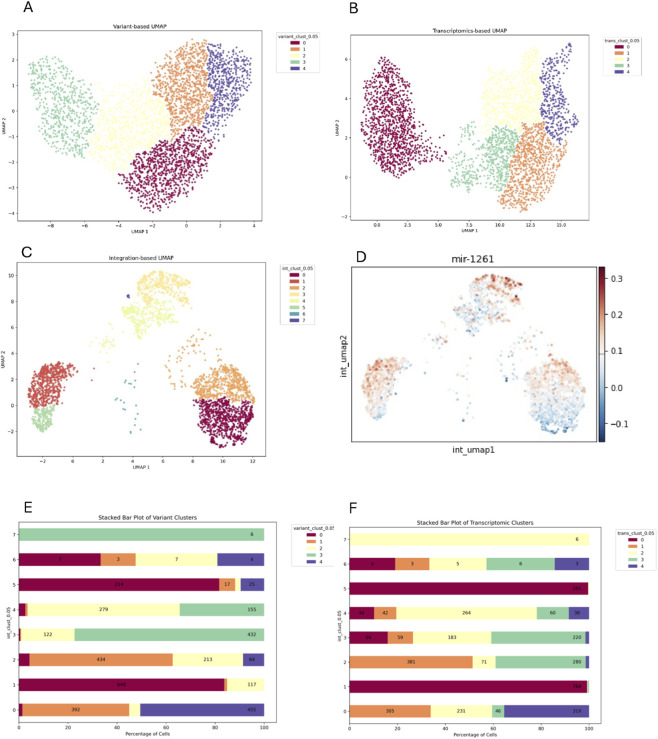
Sample L5. **(A)** Variant UMAP with cells colored by variant clusters. **(B)** Transcriptomic UMAP with cells colored by transcriptomic clusters. **(C)** Integrated UMAP with integrated clusters. **(D)** FeaturePlot showing the average expression of the mir-126 signature. Color gradient goes from blue (underexpression) to red (overexpression). The stacked barplot shows the integrated clusters distribution across variant **(E)** and transcriptomics clusters **(F)**. The y-axis represents genomics transcriptomics clusters respectively, while the x-axis shows the percentage of cells within each transcriptomics cluster, broken down by integrated clusters (indicated by different colors).

A similar situation was found in L5 sample, were both the variant and transcriptomic based clustering identified five clusters (Figures 5A,B) while integration revealed eight clusters separated into three distinct subgroups (Figure 5C). To get insights on the hidden biological meaning we computed the average expression of mir-126 signature and found that this variable could have driven the integrated clustering and may be the result of genetic and transcriptomic variability (Figure 5D). Stacked plots (Figures 5E,F) further showed that no single omics layer dominated the integrated clustering, with the exception of integrated cluster 5, which was slightly guided by transcriptomics. Similar consideration could be done based on sample L7 results. None of the two omics drove the integration, except from integrated cluster 2, which is composed mainly from cell coming from variant cluster 2 (Supplementary Figure S3).

**FIGURE 6:**
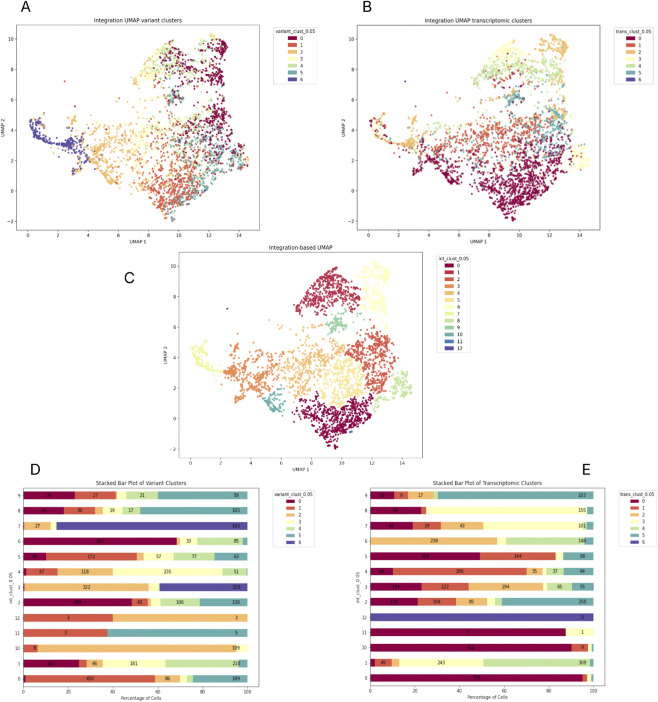
Relapse sample L4 (matched L2). Integrated UMAP with cells colored by variant **(A)**, transcriptomics **(B)** and integrated clutering **(C)**. The stacked barplot shows the integrated clusters distribution across variant **(D)** and transcriptomics clusters **(E)**. The y-axis represents genomics transcriptomics clusters respectively, while the x-axis shows the percentage of cells within each transcriptomics cluster, broken down by integrated clusters (indicated by different colors).

Among the three relapses, L4 is the only one in which the number of variant-based clusters is higher than the one at diagnosis (Figure 6A). This is not surprising, as tumor B-cells blasts could acquire new mutation leading to relapse. In this case, integration produced twelve clusters (Figure 6C), none of which was clearly driven by a single modality, suggesting genuine underlying heterogeneity. In contrast, relapse L8 was composed of a low number of cells and displayed very few detectable variants, and its integrated clustering was largely driven by transcriptomics (Supplementary Figure S4). Relapse sample L6, instead was composed by the same number of variants with respect to the matched diagnosis L5. Interestingly, scVAR integration did not give rise to highly distinguishable subgroups as in the diagnosis. Additionally, two integrated clusters (7 and 8) were mainly composed by variant cluster 4 (Supplementary Figure S5). These relapse-specific patterns collectively illustrate how clonal architecture can diverge in distinct ways across disease recurrence, with each sample capturing a different balance between mutational signals and transcriptomic organization.

Overall, these results indicate that scVAR integration can capture a broader and more informative spectrum of biological heterogeneity in B-ALL than in AML, as the unsorted nature of the B-ALL dataset preserves multiple transcriptional and genetic subtypes that remain detectable especially at diagnosis. In contrast, the mutation-selected AML dataset inherently compresses its clonal landscape, reducing the number of variant-defined structures available for integration and limiting the extent of variability that scVAR can resolve.

## 4 Discussion

Tumor heterogeneity, especially in hematologic malignancies, is a major problem for treatment response and prognosis. While single-cell RNA-seq does identify differences in gene activity, it does not completely disclose clonal architecture nor the genetic changes that drive cell identity. We developed scVAR, a method to derive and integrate genetic variants from scRNA-seq in the absence of parallel DNA sequencing. scVAR employs a variational autoencoder to generate a latent space that merges transcriptional and genetic information to more accurately describe cell populations.

In most AML and B-ALL cohorts, scVAR revealed latent substructures not apparent from transcriptomics or variant data alone. For example, in PT08-D30 AML sample, a transcriptomic HSC-like cell cluster was split into two subclones with divergent gene expression, including Myc target upregulation, suggesting biological relevance. However, relapses from patient PT08 had limited benefit from integration, suggesting that cells at relapses have a homogenous genomic variant landscape. scRNA-seq only detects variants in expressed regions, and 10 × 3′ methods mostly recover 3′ UTRs, so there is limited coverage of coding regions where functional impacting mutations can be found. Variant matrices in relapse samples were very sparse, with most sites covered in less than 1% of cells. PT08 relapse heatmaps from variant data contain many large areas of missing values, and the observed patterns could reflect coverage differences rather than genuine genotype changes. As a result, in this setting, variant-based clustering largely recovered transcriptional profiles.

Despite these limitations, scVAR was still able to delineate hidden layers of variability. In L2, scVAR allowed to identify sub-clusters dissecting original transcriptomic clusters suggesting to a novel data organization. In L5, integration consolidated the data into eight clusters instead of five, in strongly agreement with miR-126 gene expression patterns, suggesting that identified subclusters are supported by biological readout. In these cases, weak or partial signals embedded within each modality were captured more effectively when learned jointly. Results were strongly dependent on sample quality and underlying biology: in L4 relapse, scVAR revealed a more clonally heterogeneous pattern than at diagnosis. These observations indicate that scVAR performs optimally when both omics provide contrasting and informative signals, and adapts accordingly when one layer contributes minimally.

Given the scRNA-seq’s biases and limitations, scVAR still managed to unearth useful genetic signals and improved classification of cells. It produces a shared space where gene changes and gene activity are integrated in a biologically relevant manner. scVAR offers a useful method to improve scRNA-seq analysis by delineating genetic variation leveraging only transcriptome information.

Beyond leukemia, the general architecture of scVAR makes it applicable to a wide range of biological contexts. Any system in which transcriptional differences are expected to correspond—fully or partially—to underlying genetic or regulatory variation could benefit from this approach. Potential applications include solid tumors with high intra-tumoral heterogeneity, longitudinal studies tracking clonal evolution under therapy, immune-mediated disorders with mixed inflammatory and resident populations, and developmental processes in which transcriptomic transitions may be accompanied by subtle genetic or RNA-editing signatures. By learning a shared latent space from heterogeneous inputs, scVAR can serve as a flexible framework for multi-layer integration in settings where parallel DNA sequencing is impractical or where multiple sources of variability coexist.

From a computational perspective, we acknowledge that variational autoencoders introduce a higher computational cost than linear or graph-based integration methods. However, scVAR mitigates these limitations through GPU acceleration, mini-batch training, and early stopping, which keep runtime manageable even for datasets containing tens of thousands of cells. Additional optimizations—such as reducing input dimensionality prior to training, adopting lighter encoder architectures, or leveraging mixed-precision training—can further improve scalability. These considerations outline how scVAR can expand to larger datasets and more complex single-cell studies in the future.

## 5 Conclusion

We show the potential of scVAR for dissecting heterogeneity, particularly in contexts in which the mutational layer of information could have a major impact on disease biology. The main limitation is the intrinsically low genomic coverage of droplet-based platforms such as 10x Genomics Chromium, which predominantly capture the 5′ or 3′ ends of transcripts. This results in biased and incomplete variant detection: scVAR, like any method relying on genotypes inferred from scRNA-seq, is limited by what is expressed and sequenced rather than by the full mutational landscape of the cell.

Furthermore, expression of variant-bearing genes may be cell-type specific, making the presence or absence of a mutation difficult to infer in cells where the gene is not expressed. This can lead to apparent false negatives or restricted variant detection even when the underlying mutation is present. These limitations underscore the need to explore additional sequencing strategies. Full-length transcriptomic methods (e.g., Smart-seq2 or Smart-seq3) (Picelli et al., 2014; Hagemann-Jensen et al., 2020) would provide richer genomic input for scVAR by greatly expanding the number and diversity of detectable variants. Although these platforms currently lack the throughput of droplet-based systems, they would be extremely valuable in settings where the detection of driver mutations or rare clones is a priority.

Despite these challenges, scVAR provides a robust computational model for integrating heterogeneous information into a unified latent space. This ability to combine transcriptomic and genomic signals within the same dataset is particularly relevant in real-life clinical and research environments, where simultaneous multi-omic profiling may be impractical or prohibitively expensive. By exposing hidden cellular structures, refining cluster identities, and highlighting genotype-phenotype relationships, scVAR supports biological discovery in complex diseases.

Importantly, the integrative capacity of scVAR also has clear translational implications. Its unified representation is well suited for lineage tracing, monitoring clonal evolution during treatment, and identifying emerging subpopulations associated with resistance or disease progression—all key components of personalized medicine. Although additional validation and richer genomic data will further strengthen these applications, scVAR lays the foundation for computational strategies that bridge research and clinical decision-making in single-cell technologies.

## Data Availability

The scVAR tool is available at http://www.bioinfotiget.it/gitlab/custom/scvar and can be installed using the Python package manager pip. For example, it can be installed by running the command pip install scvar. This allows users to install scVAR without requiring additional configuration. The processed single-cell datasets used in this study, including AML, B-ALL and the synthetic benchmarking data, are publicly available at: https://www.dropbox.com/scl/fo/kc49b6y47hjf2zdle1zz2/AA-UA7lKpLpdHOTldAhasds>rlkey=4dkx4t5yxc407twomwqjte65panddl=0. Supplementary figures have also been included in this work.
