# Supplementary material for: scVAR: integrating genomics and transcriptomics from single-cell RNA-seq —insights from leukemia case studies
Source: Front Genet. 2026 Jan 5;16:1604484. doi: 10.3389/fgene.2025.1604484 (PMC12812400; doi:10.3389/fgene.2025.1604484)
Supplement: Supplementary file 1 [file DataSheet1.docx]

Supplementary Material

**
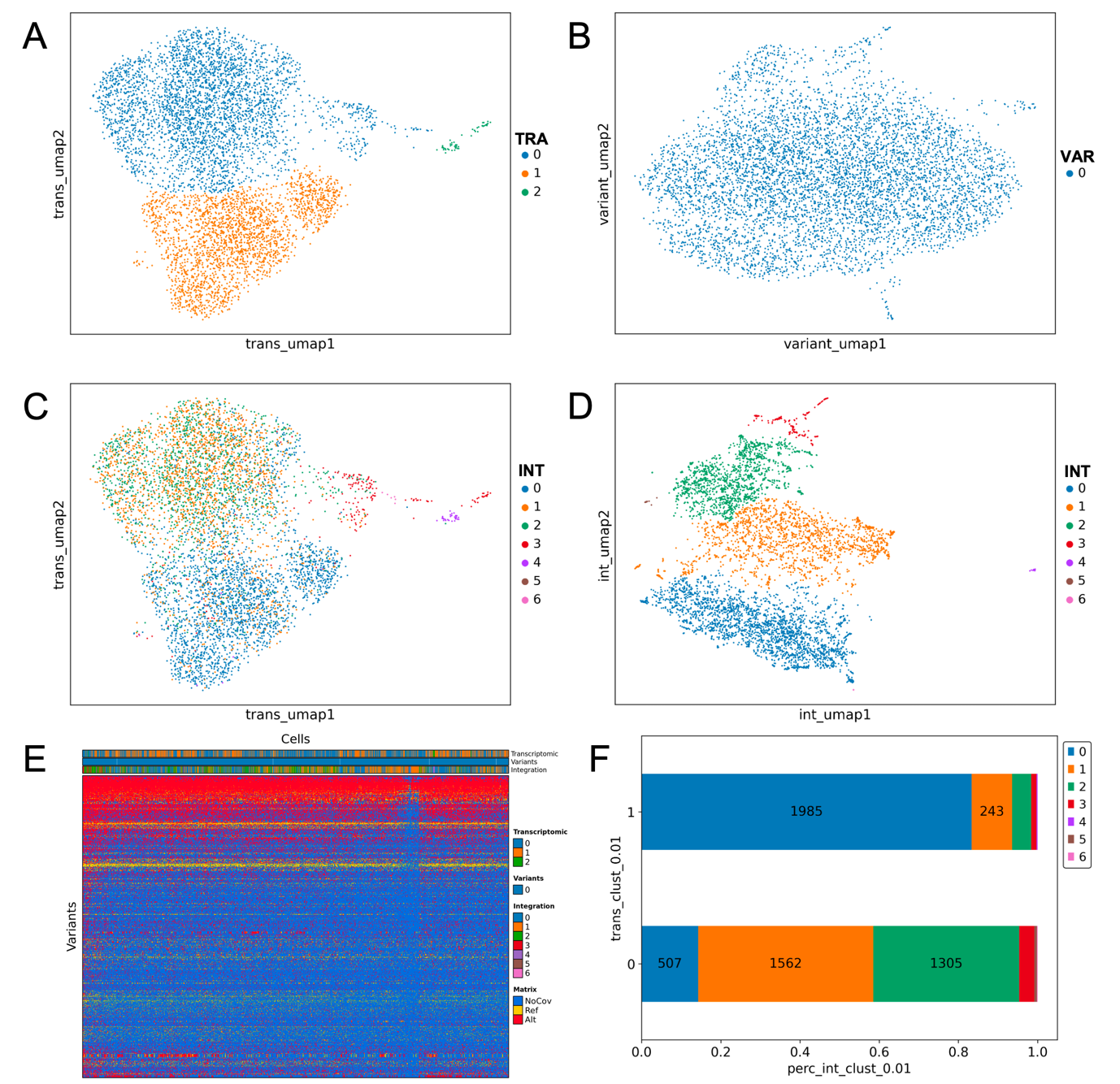
1** **Supplementary Figures**

**Supplementary Figure 1**. **Sample PT07 at D14 post-chemo.** (A) UMAP representation of transcriptomics clustering, (B) UMAP representation of variant clustering. Using the same resolution as the transcriptomics clustering, no subgroups were detected. However, by increasing the resolution, distinct clusters emerge. (C). UMAP representation of integrated clusters overlaid on the transcriptomics UMAP. (D) UMAP representation of integrated clusters in the integrated space. (E) Variant per cell matrix, with annotations for transcriptomics, variants, and integrated clusters displayed at the top. The matrix visualizes the genotypes for each cell position, with color coding as described in the legend (NoCov = no coverage variants; Ref = reference allele; Alt = Alternative allele). (F) Distribution of integrated clusters across the two main transcriptomics clusters. The y-axis represents transcriptomics clusters, while the x-axis shows the percentage of cells within each transcriptomics cluster, broken down by integrated clusters (indicated by different colors). The numbers above each bar represent the number of cells in each integrated cluster.


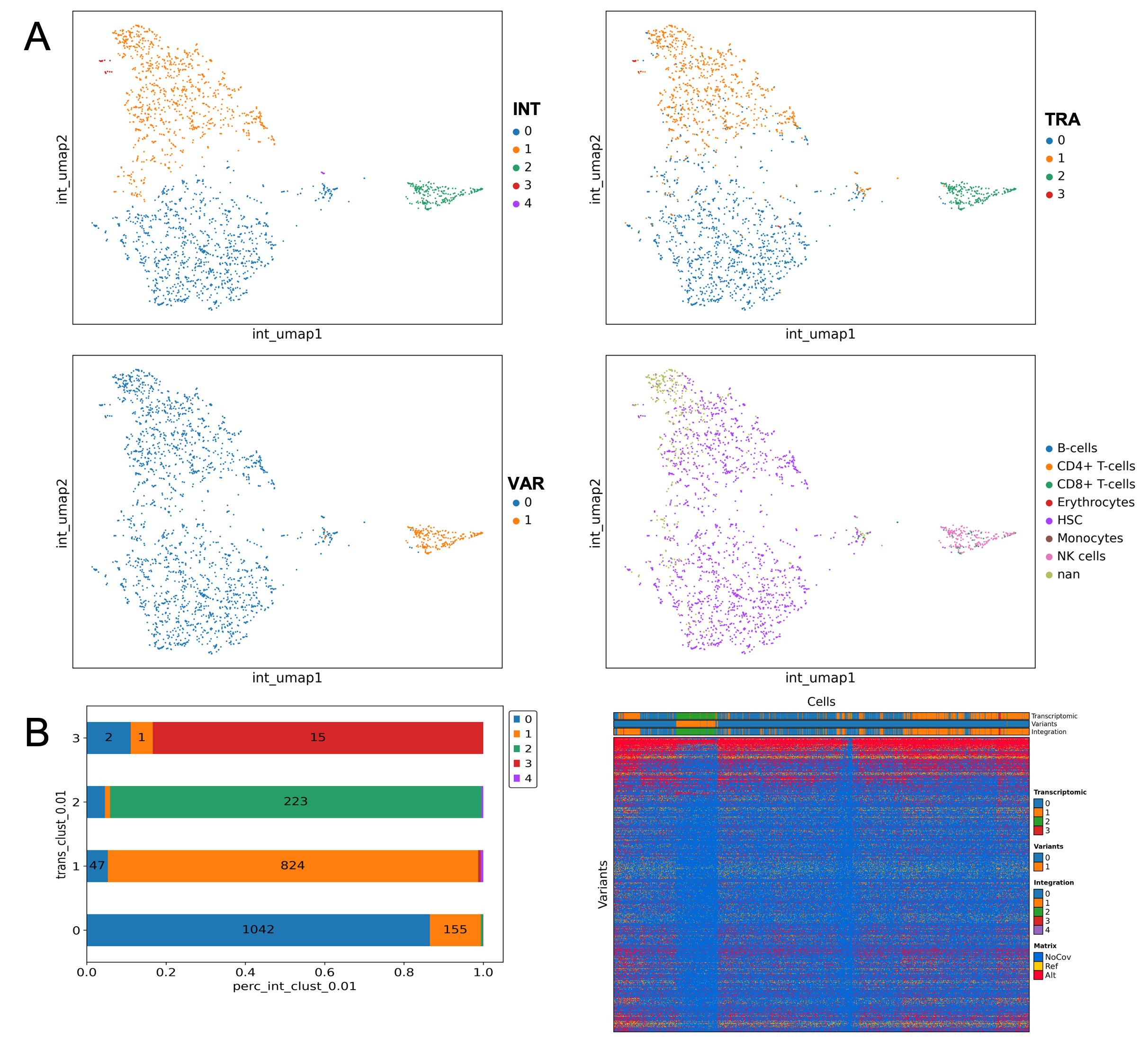


**Supplementary Figure 2**. **PT08 non-responder relapse**. (A) Integrated UMAP with cells colored by integrated, transcriptomics and variants clustering and by cell types inferred by SingleR. (B) The stacked barplot shows the integrated clusters distribution across transcriptomics clusters. The heatmap shows the distribution of clusters considering the variants coverage (NoCov = no coverage variants; Ref = reference allele; Alt = Alternative allele).

**Supplementary Figure 3. Sample L7.** Integrated UMAP with cells colored by variant (A), transcriptomics (B) and integrated clustering (C). The stacked barplot shows the integrated clusters distribution across variant (D) and transcriptomics clusters (E).

**Supplementary Figure 4. Relapse sample L8 (matched L7).** Integrated UMAP with cells colored by variant (A), transcriptomics (B) and integrated clustering (C). The stacked barplot shows the integrated clusters distribution across variant (D) and transcriptomics clusters (E).

**Supplementary Figure 5. Relapse sample L6 (matched L5).** Integrated UMAP with cells colored by variant (A), transcriptomics (B) and integrated clustering (C). The stacked barplot shows the integrated clusters distribution across variant (D) and transcriptomics clusters (E).
